# Quantifying the resolution of spatial and temporal representation in children with 22q11.2 deletion syndrome

**DOI:** 10.1186/s11689-019-9301-1

**Published:** 2019-12-20

**Authors:** Kathryn L. McCabe, Abbie M. Popa, Courtney Durdle, Michele Amato, Margarita H. Cabaral, Joshua Cruz, Ling M. Wong, Danielle Harvey, Nicole Tartaglia, Tony J. Simon

**Affiliations:** 1 Department of Psychiatry and Behavioral Sciences, University of California, Davis, CA USA; 2 MIND Institute, University of California, Davis, CA USA; 3 Division of Biostatistics, Department of Public Health Sciences, University of California, Davis, CA USA; 4 Department of Pediatrics, University of Colorado School of Medicine, Aurora, CO USA

**Keywords:** Children, 22q11.2 deletion syndrome (22q11DS), Magnitude processing, Attention, Spatiotemporal attention

## Abstract

**Objectives:**

Our ability to generate mental representation of magnitude from sensory information affects how we perceive and experience the world. Reduced resolution of the mental representations formed from sensory inputs may generate impairment in the proximal and distal information processes that utilize these representations. Impairment of spatial and temporal information processing likely underpins the non-verbal cognitive impairments observed in 22q11.2 deletion syndrome (22q11DS). The present study builds on prior research by seeking to quantify the resolution of spatial and temporal representation in children with 22q11DS, sex chromosome aneuploidy (SCA), and a typically developing (TD) control group.

**Participants and methods:**

Children (22q11DS = 70, SCA = 49, TD = 46) responded to visual or auditory stimuli with varying difference ratios. The participant’s task was to identify which of two sequentially presented stimuli was of larger magnitude in terms of, size, duration, or auditory frequency. Detection threshold was calculated as the minimum difference ratio between the “standard” and the “target” stimuli required to achieve 75% accuracy in detecting that the two stimuli were different.

**Results:**

Children with 22q11DS required larger magnitude difference between *spatial* stimuli for accurate identification compared with both the SCA and TD groups (% difference from standard: 22q11DS = 14; SCA = 8; TD: 7; *F*  = 8.42, *p* < 0.001). *Temporal* detection threshold was also higher for the 22q11DS group to both visual (% difference from standard: 22q11DS = 14; SCA = 8; TD = 7; *F*  = 8.33, *p* < 0.001) and auditory (% difference from standard: 22q11DS = 23; SCA = 12; TD: 8; *F*  = 8.99, *p* < 0.001) stimuli compared with both the SCA and TD groups, while the SCA and TD groups displayed equivalent performance on these measures (*p'*s > 0.05). Pitch detection threshold did not differ among the groups (*p*'s > 0.05).

**Conclusions:**

The observation of higher detection thresholds to spatial and temporal stimuli indicates further evidence for reduced resolution in both spatial and temporal magnitude representation in 22q11DS, that does not extend to frequency magnitude representation (pitch detection), and which is not explained by generalized cognitive impairment alone. These findings generate further support for the hypothesis that spatiotemporal hypergranularity of mental representations contributes to the non-verbal cognitive impairment seen in 22q11DS.

## Introduction

Individuals with one of a range of genetic neurodevelopmental disorders, including chromosome 22q11.2 deletion syndrome (22q11DS), Turner syndrome [1–3], Fragile X syndrome [4, 5], and Williams syndrome [6–11], show difficulties with processing visuospatial and numerical information. Explanations for these impairments vary. One explanation is that visuospatial and numerical impairment reflects general cognitive impairment, as reflected in lower IQ scores. An alternative explanation is that these impairments are the corollary of domain-general executive processing disturbances [6, 12]. A complementary hypothesis is that some specific representational or processing impairment involving spatiotemporal information results in the reduced accuracy of performance in a range of tasks into which these feed [13], causing the non-verbal cognitive impairments seen in 22q11DS and other neurodevelopmental disorders, including visuospatial and numerical ability.

22q11DS is a genetic neurodevelopmental disorder caused by a de novo deletion on the long arm of chromosome 22, and it has an estimated prevalence of 1:2000–4000 live births [14, 15]. The 22q11DS cognitive phenotype is variable, intellectual abilities are generally within the borderline range (IQ 70–84), and there is reliable evidence of less accurate and/or more variable performance on measures of attention and visuospatial cognition [16–23]. While studies have separately demonstrated evidence of difficulties in spatial and temporal attention in 22q11DS, from the existing literature, it is not clear to what extent impaired mental representations of space and time impact performance in individuals with 22q11DS. It is also unclear whether the capacity to form accurate mental representations of stimuli is limited to the spatial and temporal domains, or impacts other domains as well (e.g., the pitch characteristics of auditory stimuli).

To estimate and compare quantities of time, space, and number, perceptual and attentional systems need to interact in order to generate mental representations. These, in combination with the acquisition of numerical conceptual knowledge (i.e., what is “three”), provide a means of quantification by which values are assigned that convert continuous magnitudes into categorical units. Resolution of mental representations reflects the “minimum spacing at which attention can select individual items” [24]. Put simply, the more similar magnitudes are, the more difficulty people have distinguishing them [25]. Mental representation of magnitude obeys Weber’s Law, which states that the smallest detectable difference or just noticeable difference is proportional to the original stimulus magnitude. Numerous studies show that children, adults, and non-human primates utilize this system of quantity representation [26–28].

When attention resources fail to adequately individuate items, this produces a phenomenon referred to as “crowding.” Likened to the resolution of a digital image, Simon [13] introduced the “spatiotemporal hypergranularity” hypothesis to explain non-verbal cognitive impairment and has examined the resolution of spatiotemporal attention in neurodevelopmental disorders with this analogy in mind. This account posits that excessive crowding of sensory stimuli results in “hypergranular” (i.e., “grainier”) or lower resolution spatiotemporal mental representations that in turn generate impairment in the cognitive functions upon which such processes depend [13]. The functional implications of “spatiotemporal hypergranularity” relate to numerous cognitive difficulties that depend proximally on accurate mental representation. These include estimating time or distance, comparing two spatial or temporal magnitudes perceived via sensory systems (e.g., taller, longer duration). Such representations are also critical to the more distal (i.e., longer in terms of developmental course) process of forming accurate categorical units such as integers or minutes, meters or millions [13], since these depend on and are built upon accurate representation of simple spatial and temporal continuous magnitudes. For example, the category “five” must come to represent any five individual and distinct units that are encountered and cannot correctly be used to refer to collections of four or six units. If numerical concepts are inaccurate, the product of this inaccuracy is cognitive impairment in domains of numeracy, navigation, planning, and organization.

In 22q11DS, evidence of spatial and temporal processing impairments are reflected in difficulties compared with their typically developing peers in goal-directed spatial selective attention [19, 29], identifying and recalling spatial information [19–21], and manipulating spatial representations of objects [16, 23], as well as difficulties with basic numerical processing, representation of quantity [1, 17], and mathematics [30]. Difficulties with temporal processing are seen in selective impairment detecting change in duration, but not pitch on change detection measures (e.g., mismatch negativity [31];), as well as difficulties completing a simple finger-tapping task. For instance, Debbane and colleagues found that participants with 22q11DS displayed more variable performance reproducing the cadence of a fixed interval tone [18]. Of particular relevance to the present study, these researchers also showed that individuals with 22q11DS had poorer resolution of temporal mental representations compared with typically developing controls. In a two-tone discrimination task, participants with 22q11DS required a larger difference between stimuli to accurately detect the longer of two tones [18]. Furthermore, the authors interpreted the significant and positive correlations between these two tasks as evidence of impairment in mechanisms common to both temporal reproduction and temporal magnitude comparison. However, a recent study by Attout and colleagues [32] indicated evidence of spatial but not temporal mental representation impairment in a group of children and adults with 22q11DS when compared with both of two control groups matched on either verbal or performance IQ. Further, spatial mental representation impairment was reported only on tasks with high visuospatial demands and did not extend to tasks measuring abilities on discrete magnitude comparison. The authors interpreted these findings as evidence for differential impairment of visuospatial but not temporal mental representation. Thus, a variety of studies by different research groups [1, 16–21, 23, 29–32] provide strong evidence for specific spatial mental representation impairment though somewhat mixed evidence for temporal mental representation impairment in 22q11DS.

In the present study, we sought to identify impairments in spatial and temporal processing in a sample of children with 22q11DS as well as to quantify the degree of this impairment (i.e., their individual minimum magnitude detection threshold) relative to a group of typically developing (TD) control participants. We used an adaptive algorithm during the task to allow us to determine the minimum ratio that was required for each participant to discriminate between two magnitudes with an accuracy of 75% or greater. This minimum ratio of magnitude discrimination performance is considered a—necessarily—indirect measure of the resolution of the mental representations (spatial, temporal, frequency) generated in order to make a response selection.

To examine whether the predicted impairments are the consequence of lower resolution (i.e., hypergranular) spatiotemporal representations in 22q11DS or whether they are a consequence of general cognitive impairment, we recruited children with sex chromosome aneuploidies (SCA) as a second comparison group. SCA results from an abnormal number of X- or Y-chromosomes and includes the polysomy conditions 47,XXY (Klinefelter) syndrome in males and 47,XXX (Trisomy/Triple X) in females. Individuals diagnosed with a Klinefelter or Trisomy X syndromes tend to display average to low average full-scale IQ with difficulties concentrated in the verbal domain with non-verbal IQ typically reported in the normal range [33–36]. The addition of an SCA group allowed us to ask whether individuals with mild cognitive impairment, primarily in the domain of verbal intelligence, which is a relative strength in most children with 22q11DS, would also show lower resolution spatiotemporal representations compared with a typically developing control group. Individuals with 22q11DS tend to display the “opposite” IQ profile with scores higher on verbal IQ than non-verbal IQ [21, 37, 38]. If the SCA group exhibited similar spatiotemporal impairments to the 22q11DS group, this would suggest that performance differences were due to general cognitive impairment rather than due to differences in mental representations.

We administered several simplified psychophysics experiments to quantify the difference in spatiotemporal resolution of children with 22q11DS compared with SCA and TD control groups. We expected that, despite lower IQ in both neurodevelopmental disorder groups, spatial and temporal detection thresholds would differ between the 22q11DS and TD or SCA groups, but not between the TD and SCA groups. Furthermore, we examined whether lower (i.e., hypergranular) resolution in 22q11DS is specific to spatiotemporal representation or whether it manifests in other domains of representation. If the spatiotemporal hypergranularity hypothesis holds, we would expect that because pitch characteristics of sounds are less reliant on spatial and temporal properties, differences between the groups in terms of the resolution of mental representations would be specific to spatial and temporal, but not pitch minimum magnitude detection thresholds. Furthermore, other populations with spatial and temporal impairments resulting from brain injury (e.g., [39]) do not show similar impairments when required to discriminate between auditory pitches.

## Methods

### Participant recruitment and sample characteristics

Participants were 70 children with 22q11DS (*n* = 30 female, mean age = 11.3 years, SD ± 2.4, mean IQ = 73.3, SD ± 13.4), 46 children with SCA (47,XXX *n* = 19 or 47,XXY *n* = 27, SCA mean age = 11.1 years, SD ± 2.2, mean IQ = 95.0, SD ± 14.2) and 49 TD children (*n* = 25 female, mean age = 10.6 years, SD ± 2.2, mean IQ = 114.1, SD ± 14.2). No significant difference in mean age (*F*(2,162) = 1.2, *p* = 0.3) or gender distribution (*χ*^2^ (2) = 1.1, *p* = 0.6) was present between the groups; however, statistically significant differences between all groups were present for IQ (*F*(2,152) = 114, *p* < 0.001; 22q11DS < SCA < TD: *p*'s < 0.001) (see Table 1).

**Table 1:** Participant Characteristics

	22q11DS	TD	SCA
	(*n* = 70)	(*n* = 49)	(*n* = 46)
Age (years)			
Mean (SD)	11.3 (2.4)	10.6 (2.2)	11.1 (2.2)
Range (years)	7.7–15	7–15.1	7.2–15.3
Gender			
% Female	43	51	41
IQ			
FSIQ	73.33 (13.44)	114.05 (14.24)	94.98 (14.22)^**a,b,c^
VIQ	78.91 (13.78)	115.86 (14.73)	95.87 (13.82)
PIQ	76.73 (13.65)	109.51 (12.38)	103.00 (22.48)

*p value <  0.01; **p value <  0.001; ^a^22q11DS < SCA; ^b^SCA < TD; ^c^22q11DS < TD

Children diagnosed with 22q11DS and TD participants were recruited through the UC Davis MIND Institute, postings on online discussion groups, foundation newsletters, and by word of mouth. SCA participants were recruited through the “eXtraordinarY kids clinic” at Children’s Hospital Colorado (by author NT). Deletion of chromosome 22q11.2 was confirmed using fluorescence in situ hybridisation (FISH) or similar test, and positive 47,XXY or 47,XXX status was confirmed via review of genetic testing results. For the 22q11DS group, parents reported their child’s psychiatric diagnostic status at the time of testing. Twenty-six individuals with 22q11DS were identified as having psychiatric diagnoses including: autism spectrum disorder (ASD) (*n* = 6), attention deficit hyperactivity disorder (ADHD) (*n* = 20), generalized anxiety disorder (*n* = 10), obsessive-compulsive disorder (*n* = 4), mood disorder (*n* = 5), sensory processing disorder (*n* = 1). Sixteen individuals were currently on some form of psychiatric medication: antipsychotics: (*n* = 6), methylphenidate or other stimulants (*n* = 14), SSRIs (*n* = 3).

Exclusion criteria for children with 22q11DS included the presence of the clinical phenotype of 22q11DS *but* the absence of a 3Mb 22q11.2 deletion. Similarly, exclusion criteria for children with SCA included the presence of the clinical phenotype of either 47,XXX or 47,XXY and the absence of the chromosomal duplication. Additional exclusion criteria for all groups included the presence of a medical disorder known to affect brain function (e.g., epilepsy or hypertension), a history of head injury and moderate/severe intellectual impairment (WISC IQ < 50).

### Intellectual functioning

The Wechsler Abbreviated Scale of Intelligence (WASI [40]) (22q: *n* = 3, SCA: *n* = 5, TD: *n* = 28) or the Wechsler Intelligence Scale for Children (WISC-IV [41]) (22q: *n* = 50, SCA: *n* = 34, TD: *n* = 15) was used to assess intellectual functioning and to calculate verbal, performance, and full-scale IQ. Several participants were missing IQ data (22q: *n* = 17, SCA: *n* = 7, TD: *n* = 6).

### Stimuli, task design, and procedure

For all tasks, detection threshold was calculated as the minimum magnitude difference ratio between the “standard” and the “target” stimuli required to achieve 75% accuracy in detecting that the two are different. For example, the “standard” in the spatial task was 226 pixels in length and the initial value of the “target” was 113 pixels (i.e., 50% the size of the standard). If a child could maintain 75% accuracy when the target was 85% the size of the standard (or 192 pixels in length), but not when the target was larger, that child’s minimum magnitude detection threshold (herein referred to as “detection threshold”) would be 15%. In all tasks, participants were seated 60 cm (screen resolution 1024 × 768) from the computer screen and completed demonstration and practice items before commencing each of the tasks.

#### Spatial attention task: adaptive magnitude comparison task

Participants compared two sequentially presented stimuli (blue vertical bars in the center of the screen) and were asked to indicate which one was longer. One bar (i.e., the “standard”) remained constant in length, while the other (i.e., the “target”) varied in length according to a parameter estimation algorithm (PEST; see [42]) that used responses to trials within a four-trial block to calculate target length in the next block, with accuracy threshold set to 75%. If accuracy in the first block was at least 75%, in the next block, the difference (measured in pixels) between the target and the standard (226 pixels) was halved; otherwise, the difference between the target and standard was doubled. Stimuli order (i.e., whether the first or second stimulus was longer) varied randomly. During each trial, on a white background, the fixation point was presented at the center of screen for 2500 milliseconds (ms), followed by a vertical bar for 1500 ms with interstimulus interval (ISI) set to 1500 ms (visual mask comprised of a rectangle with gray textured hatching) that covered an area larger than the task stimuli, then the second stimulus (vertical bar) for 1500 ms. Target stimuli ranged from 113 to 224 pixels. There were a total of 24 trials (6 blocks × 4 trials per block).

#### Temporal attention task: temporal duration judgment (visual) task

Adapted from Debbané et al. [18], participants compared two sequentially presented stimuli (two pandas) and were asked to determine which was presented on the screen for longer. Fixation point was presented at the center of the screen for 1000 ms followed by two sequentially presented stimuli with ISI set to 1000 ms. The presentation duration of the standard stimulus was set to 400 ms, while the target stimulus duration was adjusted based on the PEST algorithm described above, ranging between 401 and 912 ms. The two stimuli were presented in a randomized order (i.e., whether the first or second stimulus duration was longer) for a total 36 trials (9 blocks × 4 trials).

#### Temporal attention task: temporal duration judgment (auditory) task

The temporal duration judgment-auditory (TDJ-A) task was similar to temporal duration judgment-visual (TDJ-V) task; however, in this task, visual and auditory cues were presented together, though visual stimuli were presented before auditory stimuli to ensure that participants were attending to the task prior to the onset of auditory stimuli. Participants were asked to determine which of two sounds was longer. Two 1000-Hz tones with volume set at 50 dB were presented sequentially. A fixation point was presented center of screen for 1000 ms and ISI was set to 1000 ms. The standard stimulus duration was 400 ms, while the target stimulus duration was adjusted based on PEST (range 401–912 ms). Standard and target stimuli were presented in a randomized order for a total 36 trials (9 blocks × 4 trials).

#### Auditory frequency attention task: adaptive pitch comparison task

The structure of the adaptive pitch comparison (APC) task was similar to the adaptive magnitude comparison (AMC) task; however, pitch stimuli accompanied the two visual stimuli. Participants were asked to identify which of two sequentially presented sounds was higher pitched. Two sequentially presented visual stimuli were delivered for 2000 ms each with pitch stimuli then accompanying the image for an additional 1500 ms with ISI set to 1500 ms. Visual stimuli were presented before pitch stimuli to ensure participants were attending to the task prior to the onset of pitch stimuli (total presentation time 3500 ms each). The standard pitch remained constant (1800 Hz). Target stimuli began at 1000 Hz (range 1000–1787 Hz) with a total 24 trials (6 blocks × 4 trials).

### Data analysis

Statistical analysis was carried out in R (https://www.r-project.org). Between-group differences in age and IQ were assessed using analysis of variance followed by post-hoc pairwise comparisons adjusted for multiple comparisons using FDR, while differences in gender were assessed using a chi-squared (χ^2^) test. On all tasks, individuals that did not reach ≥ 50% accuracy in discriminating magnitudes with the largest difference (referred to throughout as Block 1) were excluded from primary analysis because this level of accuracy was below chance and indicated that they were unable to perform the task. Chi-square tests or Fisher’s exact tests were used to explore between groups differences in participant’s ability to perform the first block above chance. The primary outcome measure was the participant’s level of ability to accurately detect difference between stimuli (*detection threshold*). Kolmogorov-Smirnov values and visual inspection of data distribution indicated that the AMC and APC were non-normally distributed. Cubic and squared transformations of AMC and APC data respectively were performed and linear regression analysis was conducted on all tasks. Age (months; centered to the mean), gender, and group were included in the models, unless otherwise noted. The authors concur with Dennis and colleagues [43] that IQ is not suited as a covariate in this type of study. This is because—among the reasons described at length in [43]—IQ does not meet the statistical requirements of a covariate (e.g., it is highly correlated with group membership), and the tasks used in this study adjusted task parameters based on individual performance making these measures within-individual measures. However, because our 22q and SCA groups were not matched on IQ, and because the SCA group reported mean IQ within the average range, interpretation of the role of general functional ability on spatial and temporal representation is somewhat hampered. For this reason, separate regression models were run with and without IQ. In addition, correlational analysis between IQ and the experimental measures were also conducted in order to identify patterns of association between these experimental and general functional ability measures. Finally, to reduce the likelihood of type-I error, corrections were performed for multiple comparisons using false discovery rate (FDR) [44].

## Results

### Adaptive magnitude comparison (AMC)

**Fig. 1:**
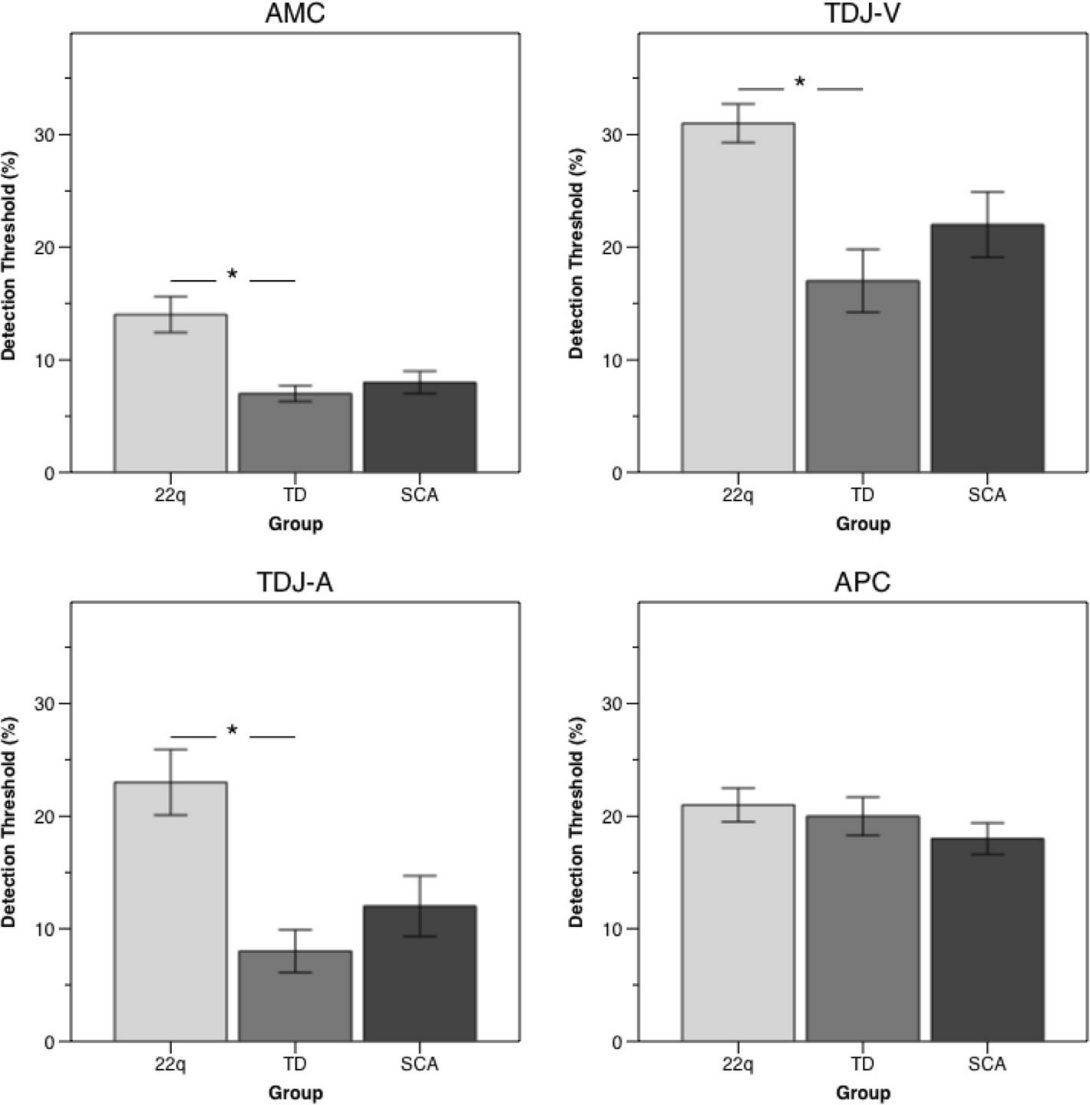
Group means and standard deviations (SD) for detection threshold for the AMC, TDJ-V, TDJ-A, and APC tasks. *p < 0001

Block 1 accuracy between the groups indicated significantly more below chance responses from children with 22q11DS compared with children with SCA and the TD group (*p* = 0.019 Fisher’s exact test; 22q11DS: *n* = 10/70 (14%); SCA: *n* = 3/46 (7%)). These participants (*n* = 13) were omitted from the primary analysis. Age and gender did not contribute to the linear regression (cube transform) model and were removed. The final model indicated that children with 22q11DS displayed a significantly higher detection thresholds for spatial magnitude (pixels) than children in the TD and SCA groups (detection threshold (%): 22q11DS = 14; SCA = 8; TD = 7; *F* (2,149) = 8.42, *p* < 0.001). Pairwise comparisons showed threshold detection differences between the 22q11DS and TD groups (*t* = − 3.8(92.5), *p* < 0.001, *d* = 0.74) and 22q11DS and SCA groups (*t* = − 2.88(99.6), *p* = 0.015, *d* = 0.58) but not the SCA and TD groups (*p* = 0.52). Mean group detection thresholds are displayed in Table 2 and Fig. 1.

**Table 2:** Group means and standard deviations (SD) for detection threshold for the AMC, TDJ-V, TDJ-A, and APC tasks

Task	*n*	Detection threshold	
		Mean	Range
AMC			
22q11DS	60	14 (12.4)	2–48^c**^
TD	49	7 (5.2)	2–27
SCA	43	8 (6.5)	2–28
TDJ-V			
22q11DS	36	31 (10.5)	12–49^c**^
TD	30	17 (15.4)	1–46
SCA	23	22 (13.8)	1–49
TDJ-A			
22q11DS	40	23 (18.3)	1–50^c**^
TD	30	8 (10.2)	1–42
SCA	27	12 (13.9)	1–50
APC			
22q11DS	59	21 (11.5)	11–50^NS^
TD	44	20 (11.3)	11–46
SCA	42	18 (8.9)	11–38

*p value <  0.01; **p value < 0.001; ^a^22q11DS < SCA; ^b^SCA < TD; ^c^22q11DS < TD; *NS*, not significant

### Temporal duration judgment-visual (TDJ-V)

Block 1 accuracy showed significantly more below chance responses from children with 22q11DS (*p* = 0.01 Fisher’s exact test; 22q11DS: *n* = 18/54 (34%); SCA: *n* = 3/26 (10%); TD: *n* = 1/31 (3%)). These were identified and omitted from primary analysis (*n* = 22). Data were normally distributed and since age and gender did not contribute to the regression model they were removed (*p*s > 0.05). The final model showed that children with 22q11DS had a higher detection threshold when comparing visual duration (ms) than the SCA and TD groups (detection threshold (%): 22q11DS = 31; SCA = 22; TD = 17; *F* (2,86) = 8.33, *p* < 0.001). Pairwise comparisons showed differences between 22q11DS and TD groups (*t* = − 4.04(48.9), *p* < 0.001, *d* = 0.87) and 22q11DS and SCA groups (*t* = − 2.31(35.9), *p* < 0.04, *d* = 0.53) but not the SCA and TD groups (*p* = 0.14) (see Fig. 1).

### Temporal duration judgment-auditory (TDJ-A)

Block 1 accuracy showed that significantly more of the children from the 22q11DS and SCA groups were unable to detect pitch duration above chance (*p* = 0.004 Fisher’s exact test; 22q11DS: *n* = 14/54 (26%), SCA = 2/29 (7%)). These participants were excluded from primary analysis (*n* = 16). Data were normally distributed. Overall, the group but not age or gender significantly contributed to the regression model and children with 22q11DS showed higher auditory duration (ms) detection thresholds compared with children in the TD and SCA groups (detection threshold (%): 22q11DS = 23; SCA = 12; TD = 8; *F* (2,94) =8.99, *p* < 0.001). Pairwise comparisons indicated detection threshold differences between children with 22q11DS and TD (*t* = − 4.057(65.9), *p* < 0.0001, *d* = 0.89); and children in the 22q11DS and SCA groups (*t* = − 2.53(63.65), *p* = 0.03, *d* = 0.61), but not SCA and TD groups (*t* = − 1.058(49.7), *p* = 0.29, *d* = 0.28) (see Fig. 1).

### Adaptive pitch comparison (APC)

Block 1 completion rate in pitch comparison did not differ between the groups (*χ*^2^(2) = 1.59, *p* = 0.45; 22q11DS: *n* = 11/70 (16%); SCA: 4/46 (9%); TD: 4/48 (8%)). These participants (*n* = 19) were omitted from the primary analysis. Linear regression (square transform) indicated that neither age, gender, nor group membership influenced pitch (Hz) detection threshold (detection threshold (%): 22q11DS = 21; SCA = 18; TD = 20, *p*s > 0.05) (see Fig. 1).

### Associations with measures of intellectual functioning (IQ)

In order to explore the contribution of IQ to spatial and temporal representation, we ran separate regression models with IQ included as a predictor for each of the measures (AMC, APC, TDJ-A/V). Age and gender did not contribute to any of the regression models and were removed.

#### AMC

Group (22q11DS: *t* = 2.3, *p* = 0.02; *t* = 2.2, *p* = 0.03) and IQ (*t* = 3.0, *p* = 0.003) contributed significantly to the model showing that children with 22q11DS *and* SCA displayed significantly higher detection thresholds for spatial magnitude (pixels) than children in the TD group (*F*(5,120) = 4.95, *p* < 0.001). IQ was also significant for all groups, i.e., detection threshold decreased with increasing IQ scores (*t* = 2.997, *p* = 0.003). Finally, interaction effects indicate that this increase in performance with higher IQ was less profound in both the groups with 22q11DS and SCA compared with the TD group (22q11DS: *t* = − 2.8, *p* = 0.005; SCA: *t* = − 2.1, *p* = 0.04).

#### TDJ-A/V

When IQ was added as a predictor to both the TDJ-A and TDJ-V models, none of the individual predictors contributed significantly to the model (*p*'s > 0.05) (TDJ-A: *F* (5,72) = 4.9, *p* < 0.001; TDJ-V: *F* (5,65) = 3.6, *p* = 0.006).

#### APC

Group (*t* = 2.3, *p* = 0.005) and IQ (*t* = 2.9, *p* = 0.005) contributed significantly to the regression model (*F* (5,111) = 3.4, *p* = 0.006). Children with SCA, but not those with 22q11DS, showed significantly lower pitch detection thresholds compared with children in the TD group.

#### Correlations with IQ

To further explore the potential contribution of IQ to task performance correlational analysis of IQ (PIQ, VIQ, and FSIQ) and measures of spatial, temporal and frequency detection were completed. These show different patterns of association among the groups (22q11DS, SCQ, TD) (see Table 3). To aid interpretation of the data—for all measures, data have been reversed so that positive correlations indicate association between lower detection threshold and higher IQ. For children with 22q11DS, significant, moderate, positive correlations between measures of spatial (AMC) and temporal (TDJ-A) representation and PIQ were reported (except for TDJ-V which instead showed significant moderate positive correlation with FSIQ and VIQ). Pitch detection was associated with all measures of IQ (PIQ, VIQ, and FSIQ) in the 22q11DS group.

**Table 3:** Correlations between measures of magnitude comparison and IQ

	22q			TD			SCA		
	FSIQ	VIQ	PIQ	FSIQ	VIQ	PIQ	FSIQ	VIQ	PIQ
AMC	0.16 (0.27)	0.15 (0.31)	0.39 (0.007)	0.65 (0.001)	0.61 (0.001)	0.48 (0.001)	− 0.02 (0.9)	− 0.05 (0.77)	0.06 (0.74)
APC	0.38 (0.01)	0.39 (0.01)	0.38 (0.01)	0.4 (0.01)	0.30 (0.06)	0.39 (0.01)	0.03 (0.85)	− 0.13 (0.44)	− 0.10 (0.56)
TDJ-A	0.3 (0.10)	0.29 (0.11)	0.58 (0.001)	0.28 (0.16)	0.17 (0.4)	0.31 (0.12)	0.41 (0.07)	0.4 (0.08)	0.15 (0.53)
TDJ-V	0.45 (0.02)	0.41 (0.04)	0.28 (0.15)	0.25 (0.20)	0.27 (0.17)	0.15 (0.44)	− 0.03 (0.92)	− 0.02 (0.93)	− 0.22 (0.4)

Nb, To aid interpretation of the data—for all measures, data have been reversed so that positive correlations indicate that lower detection threshold is associated with higher IQ

Interestingly, we saw no association between IQ and representation measures by the SCA group. For the TD group, different patterns again were reported—spatial detection (AMC) was significantly positively correlated with all measures of IQ (PIQ, VIQ, FSIQ), while frequency detection (APC) was associated with FSIQ and PIQ, but not VIQ. No association was observed between IQ and temporal representation measures (TDJ-A, TDJ-V).

## Discussion

We sought to quantify the resolution of spatiotemporal representation and to compare performance among two groups of children with neurodevelopment disorders (22q11DS and SCA) and TD controls. Using tasks designed to examine spatial and temporal magnitude estimation, we replicated and extended previous findings reported by Debbané [18] and Simon [1, 17] demonstrating in children with 22q11DS increased sensitivity to the distance effect, whereby participants with 22q11DS required larger differences (i.e., higher detection threshold) to accurately compare magnitude in both visual and auditory modalities. Participants were required to determine difference between sequentially presented spatial, temporal, and pitch stimuli in a series of simple comparison tasks. We interpret these findings as evidence of reduced resolution in the mental representations needed to quantify space and time and heightened vulnerability to attentional crowding. Or in terms of our digital imaging analogy, the resolution of spatial and temporal representations in children with 22q11DS was “grainier” than those of TD children and those with SCA. As expected, these impairments were specific to spatial and temporal domains, as no group differences were observed in pitch detection thresholds. This indicates that group differences in the spatial and temporal tasks are not due to inability to perform comparison tasks in general and supports the hypothesis that cognitive impairment of attention resolution in 22q11DS is preferentially sensitive to the spatiotemporal properties of stimuli and does not extend to impairment of the mental representation of frequency (pitch).

It is important to note that compared with age- and gender-matched TD peers and those with SCA, more children with 22q11DS (between 14 and 34%, depending on the task) than those with SCA or TD children (0–10%) were unable to perform each task. That is, they were unable to perform above chance when the magnitude difference between stimuli was largest. These children may represent a sub-group of children within the larger 22q11DS sample who present with severe impairment in magnitude comparison. However, it is important to note that when examined on a case-by-case basis, the severe impairment sub-group was not comprised of the same participants across the different spatial, temporal, and pitch comparison tasks.

### Quantifying the resolution of magnitude representation in 22q11DS and SCA

A significant difference in detection threshold between the TD group and children with 22q11DS and between the SCA group and children with 22q11DS clearly showed that children with 22q11DS needed the differences between the spatial and temporal extent of the standard and target bars to be significantly larger than was required for the children in the TD and SCA groups in order to be able to perform at the same level of accuracy (i.e., 75%). Since our psychophysics-like tasks held performance constant and adjusted detection thresholds, our results allow us to produce the first estimates of the “degree of hypergranularity,” or severity of impairment causing reduction in resolution of spatial and temporal representations that is experienced by our sample of children with 22q11DS. As expected, the SCA group displayed comparable performance to the TD group on tasks of spatial, temporal, and pitch detection. On the spatial resolution task, given the smallest standard/target difference for TD children was 7% and the smallest ratio for children with SCAs was 8%, the 14% ratio for our 22q11DS group suggests that their mental resolutions for spatial extent were 6–7% less complete than was true for the other groups.

Overall, all groups demonstrated poorer temporal magnitude detection threshold compared with the spatial magnitude threshold. On the temporal detection tasks, participants reported higher detection thresholds and more participants were excluded for failing to respond above chance. Given the smallest standard/target ratio for TD children on the temporal duration judgment was between 8% (auditory) and 17% (visual), and the smallest ratio for children with SCAs was between 12% (auditory) and 22% (visual), a standard/target ratio between 23% (auditory) and 31% (visual) for our 22q11DS group suggests that their mental resolutions for spatial extent were between 9 and 15% less complete than was true for the other groups. Further, it is important to note that the 22q11DS group performed comparably with both SCA and TD groups on the pitch detection task (ratio: 22q = 21%; SCA = 18%; TD = 20%).

Though methodological differences prevent direct comparison between studies, our findings are consistent with those reported by Debbané et al. [18] on measures of temporal magnitude discrimination—they also showed that children with 22q11DS required larger difference between stimuli to accurately detect difference in tone duration, but not pitch. Surprisingly, given the similarity in some aspects of task design and presentation, a recent study by Attout et al. [32] did not replicate the findings reported Debbané and colleagues [18] or those of the present study that showed temporal magnitude representation impairment in individuals with 22q11DS. Participant characteristics such as, age range, or differences in methodology and accuracy analysis may account for the difference in findings.

Our findings indicate that processes related to forming frequency-based mental representation and detecting difference between auditory pitches is equivalent in 22q11DS to children with SCA and TD peers. These findings show that properties of the representation of spatial and temporal stimuli that are not found in pitch stimuli are specifically impaired in 22q11DS, and that performance on these tasks is not the secondary consequence of task design. Further, we interpret these findings to indicate that children with 22q11DS present with specific impairment in spatiotemporal magnitude estimation that does not extend to pitch detection.

### 22q11DS cognitive phenotype: evidence for selective spatiotemporal impairment

As mentioned previously, the broad cognitive phenotype of 22q11DS is characterized by impairment in the non-verbal domain [21, 37, 38]. Moreover, previous literature point to impaired representation of space, time, and number in 22q11DS, with difficulties documented on measures of numeracy, visuospatial processing [17, 19], spatial working memory, and executive functioning [17, 21, 45]. The present study extends previous research in this area by explicitly quantifying spatiotemporal representational abilities in 22q11DS compared with TD children and children with SCA.

A methodological strength of the present study is the addition of the pitch detection paradigm (APC) with identical PEST algorithm and design to the spatial magnitude comparison task (AMC). Thus, if task performance was the consequence of general working memory and/or attention impairment, we could reasonably expect similar patterns of performance across tasks. However, as stated above, when compared with age- and gender-matched children with a different NDD (SCA) or the TD group, children with 22q11DS demonstrated comparable ability to generate mental representations of frequency estimation to discriminate between tones. Thus, the cognitive impairment associated with 22q11DS does not impede the ability to represent and compare frequency information.

### Spatiotemporal hypergranularity hypothesis

The profile of performance reported here by children with 22q11DS is consistent with what would be expected from a developmental impairment in spatiotemporal mental representation and relatively intact mental representation of pitch. This hypothesis was tested with the spatial magnitude comparison task, temporal duration tasks (visual and auditory versions), and a pitch comparison task. This study advanced the hypothesis of specific spatiotemporal hypergranularity in 22q11DS, first by demonstrating evidence of reduced resolution of spatial and temporal mental representations on a series of simple psychophysics comparison tasks, and secondly by demonstrating uneven magnitude estimation performance across sensory domains, demonstrating relatively intact pitch perception abilities compared with children with SCA and TD controls. Thus, impairment was specific to spatiotemporal task demands and was not accounted for by more general attentional or working memory aspects of the task design.

### Limitations

Before we address the potential contribution of intellectual impairment on task performance, there are several limitations associated with the current study. These relate primarily to task characteristics as well as the measurement of IQ. It should be noted that the number of trials in each task was relatively small compared with typical psychophysiology studies. While this was an intentional methodological decision to minimize participant fatigue, replication with additional trials is recommended. However, similarities between our findings and previous studies utilizing similar paradigms (e.g., [18]) coupled with adequate power would suggest that the reduced number of trials did not adversely impact our results. Secondly, rates of excluded children with 22q11DS were unexpectedly high on the temporal duration judgment (TDJ-A/V) tasks (auditory: 22q11DS: 26% (*n* = 14/53), visual: 22q11DS: 34% (*n* = 18/53)). While worth noting, this outcome may not necessarily represent a methodological weakness. Results showed, across all groups, the same pattern of poorer performance on the TDJ-A/V tasks. Therefore, while children with 22q11DS demonstrated impaired performance, the threshold of > 50% accuracy for inclusion in the primary analysis indicated that participants included in analysis understood the requirements of the task. Furthermore, it does not appear that task difficulty explains the TDJ-A/V exclusion rates in the 22q11DS group. We report that only one TD participant failed to meet block 1 accuracy thresholds for these tasks compared with *n* = 4 (8%) of TD participants who failed to meet block 1 threshold on the APC task.

The primary limitation of this study relates to several aspects to do with the measurement and treatment of IQ. Participants completed either the 4-subscale WASI-2 or the more comprehensive WISC-IV assessment. Although the comparability of these measures is acceptable, with correlation coefficients of 0.82 (PRI), 0.85 (VCI), and 0.91 (FSIQ) respectively, this may have resulted in slightly higher WASI scores compared with their WISC-IV equivalent. Another limitation to the study relates to IQ and the NDD groups. In addition to characterizing spatial and temporal representation abilities in a group of children with SCA, the inclusion of an SCA group was intended to address several methodological matters common to neurodevelopmental disorders research. Matching samples on IQ runs the risk that IQ-matched group heterogeneity will impede meaningful comparisons, while controlling for features (such as IQ) that are highly correlated with group membership creates similar impediments to interpreting findings. Thus, in the present study, we recruited participants from a cohort with a defined chromosomal duplication with mild intellectual impairment and a cognitive profile “opposite” to 22q11DS. Though statistically different from both the 22q11DS and TD groups—the SCA group had a mean FSIQ within the average range. Thus, our NDD contrast group was not FSIQ matched to our 22q11DS group and did not present with mild intellectual impairment at the group level. Furthermore, our 22q11DS group did not display relative strengths in VIQ and members of our TD control group had FSIQ well above average. Together, these factors limited our interpretation of the data in relation to the effect of IQ on task performance.

For these reasons, we chose to run analysis with and without IQ included as a predictor in the regression model, and performed correlational analysis to examine the patterns of association of general functional ability (IQ) and our experimental measures. It is our contention that IQ is a group-defining characteristic of 22q11DS. Moreover, we consider IQ largely unsuited as a covariate in neurocognitive studies of individuals with NDDs (see [43] for a nuanced discussion of the topic). To summarize, the authors argue that IQ does not meet the requirements of a covariate, and incorporating IQ into analysis of NDDs risks “overcorrected, anomalous, and counterintuitive findings about neurocognitive function” ([43], pp331). However, as the SCA group FSIQ fell within the average range, the contribution of general cognitive ability to task performance remained unclear. When IQ was included as a predictor, we saw several changes to our findings—first, for temporal measures (TDJ-A/V), none of the model predictors remained significant. For spatial representation (AMC), findings shifted to include significant group differences between the SCA and TD groups, as well as a significant contribution of IQ to the model. While for the pitch detection task, we showed a group effect, but that only the SCA group differed from TD children on this measure. IQ significantly contributed to the model, but did not do so differentially for the three groups (unlike its pair AMC). In addition, correlational analysis confirmed these different patterns of association between IQ and the experimental measures among the groups. Given the similarity in task design and demands, if performance was explained—at least in part—by IQ, we would expect the effects of IQ to be reasonably stable between the tasks and groups. The SCA and TD groups both reported FSIQ in the average range and with similar variance in performance, yet we see marked difference in correlation of IQ and experimental measures. The SCA group performance on these measures shows no association with IQ, while the TD group reports fairly consistent associations with some measure of IQ for magnitude and frequency, but not temporal detection. Therefore, based on these combined findings and previous reports on this topic, we contend that the model excluding IQ is the more parsimonious of the two models, and as such, we consider these the basis of our key findings in this study. However, we must acknowledge the complex role that IQ may be playing in our findings. Future studies will help to clarify its involvement through recruitment of NDD contrast groups that are a closer match on IQ.

A final important consideration regards spatiotemporal representation causing downstream difficulties in non-verbal cognition in 22q11DS. We see some support for this hypothesis. Higher PIQ was associated with lower detection threshold for three of the four measures (AMC, APC, TDJ-A). It is difficult to explain why PIQ would be associated with the auditory measure of temporal representation but not the visual. Participants with 22q11DS reported poorest and least variable performance on TDJ-V; however, though it was not associated with PIQ, better performance was significantly positively correlated with FSIQ and VIQ on this measure in the 22q11DS group only.

Methodological strengths of the study relate to the inclusion of age- and gender-matched TD and SCA groups as well as the addition of the pitch detection task that allowed us to explore the specificity of spatiotemporal representation impairment in these groups. Finally, the application of PEST instead of reaction time measures, which are more typical to psychophysical and behavioral paradigms, allowed us to quantify each individual’s detection threshold. Reaction time is notably heterogeneous, often age-dependent, and not normally distributed in neurodevelopmental disorders. Thus, the PEST method allowed us to more directly measure the cognitive feature that we were most interested in (i.e., detection threshold) rather than an indirect manifestation of detection threshold (e.g., reaction time).

### Clinical implications

Clinical implications primarily relate to the potential for remediation of the cognitive processes underpinning spatiotemporal representation. Specifically, we will be interested to know whether targeted training can improve spatiotemporal resolution; whether improvements generalize to related cognitive skills; what is the optimal “dose” of cognitive training required to yield improved performance; and importantly, whether training can translate to improve functioning in everyday life.

## Conclusions

The elevated thresholds for spatial and temporal but not pitch representation indicate that neither working memory load nor general attention impairment adequately accounts for the pattern of performance by the 22q11DS group, and that these impairments relate specifically to the spatial and temporal characteristics of the stimuli. Furthermore, these difficulties were not observed in the SCA group, which suggests that spatiotemporal processing difficulties do not generalize to all neurodevelopmental disorders. Finally, we conclude that in the current study, spatiotemporal impairment is not a corollary of general working memory or attention impairment and is a promising opportunity for targeted remediation.

## Data Availability

Requests for de-identified data reported in the study will be considered by the authors. Interested researchers should contact the corresponding author with their request.

## Abbreviations

22q11DS: Chromosome 22q11.2 deletion syndrome
47,XXX: Trisomy X
47,XXY: Klinefelter Syndrome
ISI: Interstimulus interval
SCA: Sex chromosome aneuploidy
TD: Typically developing
